# T1 mapping in Alzheimer’s disease: a quantitative MRI study with amyloid-PET correlation

**DOI:** 10.1186/s41747-026-00792-1

**Published:** 2026-09-21

**Authors:** Lorenzo Gualco, Noemi Montobbio, Mattia Losa, Mauro Costagli, Federico Massa, Beatrice Orso, Lucia Argenti, Enrico Peira, Andrea Chincarini, Silvia Morbelli, Stefano Raffa, Luca Sofia, Gianmario Sambuceti, Luigi Lorenzini, Emanuele Pravatà, Massimo Caulo, Martina Pulze, Dario Arnaldi, Andrea Diociasi, Domenico Zacà, Gian Franco Piredda, Tom Hilbert, Tobias Kober, Marco Bozzali, Maria Pia Sormani, Matteo Pardini, Luca Roccatagliata

**Affiliations:** 1 Department of Neuroscience, Rehabilitation, Ophthalmology, Genetics, Maternal and Child Health (DINOGMI), University of Genoa, Genoa, Italy; 2 IRCCS AOU SS Antonio e Biagio e Cesare Arrigo, Alessandria, Italy; 3 Department of Health Sciences (DISSAL), University of Genoa, Genoa, Italy; 4 AOM – IRCCS Ospedale Policlinico San Martino, Genova, Italy; 5 National Institute of Nuclear Physics (INFN) Genoa Section, Genoa, Italy; 6 Nuclear Medicine Unit, Città della Salute e della Scienza di Torino, Turin, Italy; 7 Department of Medical Sciences, University of Turin, Turin, Italy; 8 Department of Radiology and Nuclear Medicine, Amsterdam University Medical Centre, Vrije Universiteit, Amsterdam, The Netherlands; 9 Department of Neuroscience, Imaging and Clinical Sciences, “G. d’Annunzio” University of Chieti-Pescara, Chieti, Italy; 10 Institute for Advanced Biomedical Technologies (ITAB), “G. d’Annunzio” University of Chieti-Pescara, Chieti, Italy; 11 Department of Internal Medicine and Medical Specialties (DIMI), University of Genoa, Genoa, Italy; 12 Scientific Collaborations and Strategic Partnerships, Siemens Healthcare S.r.l., Milan, Italy; 13 Swiss Innovation Hub, Siemens Healthineers International AG, Lausanne, Switzerland; 14 Ecole Polytechnique Fédérale de Lausanne (EPFL), Lausanne, Switzerland; 15 Department of Radiology, Lausanne University Hospital and University of Lausanne, Lausanne, Switzerland; 16 Research & Clinical Translation, Magnetic Resonance, Siemens Healthineers AG, Erlangen, Germany; 17 ‘Rita Levi Montalcini’ Department of Neuroscience, University of Torino, Turin, Italy; 18 SC Neurologia 2U, AOU City of Health and Science, Turin, Italy

**Keywords:** Alzheimer disease, Biomarkers, Cognitive dysfunction, Magnetic resonance imaging, Positron-emission tomography

## Abstract

**Objectives:**

Quantitative T1 mapping (qT1) is a magnetic resonance imaging (MRI) biomarker of brain microstructural changes; however, its application in Alzheimer disease (AD) remains limited. We compared cortico-limbic qT1 values between patients with AD and healthy controls (HCs) and examined their relationship with amyloid burden measured by amyloid-positron emission tomography (PET).

**Materials and methods:**

We retrospectively identified subjects with mild cognitive impairment due to AD and HCs who underwent 3-T MRI, including a compressed-sensing MP2RAGE sequence, generating three-dimensional T1-weighted images and qT1 maps. Amyloid-PET was available for 16 patients. Hippocampal qT1 and volume were compared between groups, adjusting for age and sex; analyses were repeated after stratifying patients by Mini-Mental State Examination (MMSE). Voxel-wise group comparison was performed using SPM 12. The qT1-standardized uptake value ratio (SUVr) association was assessed with a linear mixed-effects model applied to voxel-level data, accounting for partial volume effects.

**Results:**

Thirty-three AD subjects, aged 69.3 ± 8.2 years (mean ± standard deviation), 18 females, and 22 HCs aged 70.5 ± 13.3 years, 15 females, were evaluated. Regional hippocampal qT1 was higher in AD (1,398 ± 53.0 ms *versus* 1,349 ± 53.4 ms; *p* = 0.002), without difference across cognitive subgroups stratified by MMSE. AD subjects exhibited increased qT1 in mesial temporal gray matter and temporo-parieto-occipital cortices (1,448 ± 55.7 ms *versus* 1,344 ± 51.6 ms; *p* < 0.001). Voxel-level modelling revealed a positive association between qT1 and amyloid-PET SUVr (*p* < 0.001; *d* = 0.223).

**Conclusion:**

qT1 mapping can be sensitive to changes related to brain amyloidosis, supporting its role as a promising, noninvasive imaging biomarker in AD.

**Key Points:**

***Question*** Can qT1 mapping detect AD-related microstructural changes in the cortico-limbic gray matter?

***Findings*** Participants with AD showed increased cortico-limbic qT1 values. A subtle yet consistent positive association was observed between cortical qT1 values and amyloid-PET SUVr.

***Relevance statement*** qT1 mapping captures microstructural tissue alterations related to amyloid pathology in Alzheimer’s disease, supporting its role as a potential non-invasive imaging biomarker in this condition.

**Graphical Abstract:**

**Figure:**
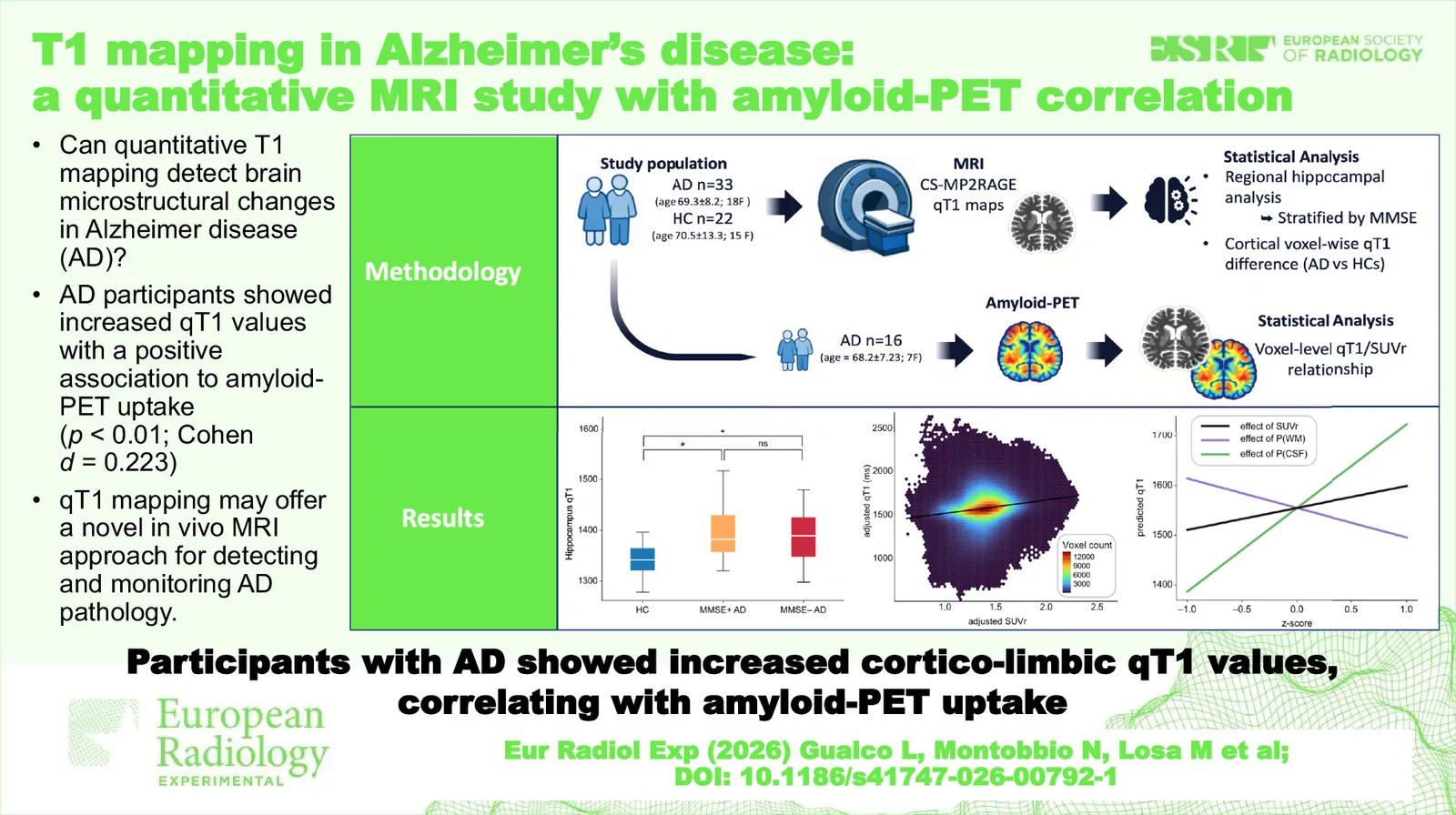

## Background

The validation of magnetic resonance imaging (MRI) biomarkers capturing key pathological changes in Alzheimer disease (AD) remains an unmet need. Atrophy represents a late-stage phenomenon, impacted by both misfolded proteins and co-pathologies. Therefore, the diagnostic workup of suspected AD requires more accurate indices of pathology, such as fluid and molecular imaging biomarkers to assess amyloid accumulation and ongoing neurodegeneration [1].

Quantitative T1-mapping (qT1) has demonstrated the ability to detect brain tissue alterations in various pathological conditions, such as multiple sclerosis [2], epilepsy [3], and brain tumours [4, 5]. The application of qT1-mapping in neurodegenerative disorders has gained increasing attention, although its role in this field remains to be fully defined, especially in AD [6].

The T1 relaxation time is influenced by tissue microscopic composition, particularly by the iron content, myelin, and macromolecular fraction [7]. Notably, T1 mapping is commonly acquired in cardiac MRI [8], where myocardial amyloid accumulation is associated with native T1 prolongation [9]. In the myocardium, T1 values are considered particularly influenced by the extracellular space volume, which is expanded in amyloidosis, also enabling a quantitative assessment of its expansion [8, 9].

Thus, the hypothesis that this metric can detect alterations typical of AD is well-founded. Evidence from previous studies indicates altered brain T1 relaxation times in AD, despite methodological heterogeneity and variable results [6, 10, 11]. This work investigates gray matter (GM) qT1 values in AD obtained with a fast acquisition method and explores their relationship with the underlying pathology as detected by amyloid- positron emission tomography (PET).

## Materials methods

### Participants

We retrospectively enrolled patients with mild cognitive impairment due to AD diagnosed according to the National Institute on Aging-Alzheimer’s Association criteria [12] and identified from an in-house database, who underwent 3-T MRI between November 2020 and April 2024. Inclusion criteria were: symptoms lasting less than two years at the diagnosis; positivity for amyloidosis biomarkers, including core cerebrospinal fluid (CSF) biomarkers (*i.e*., amyloid-β isoforms, tau protein species, and their clinically validated ratios), or amyloid-PET; unchanged diagnosis during the follow-up; no prior psychiatric, neurological, or major medical disorders. Healthy controls (HCs) were recruited among non-blood relatives of patients and by word-of-mouth. Their cognitive status was checked with a Mini-Mental State Examination (MMSE) score > 27 and a Clinical Dementia Rating scale score = 0.

Demographic features were compared between patients and HCs with the Mann–Whitney *U*-test and Pearson χ^2^ as appropriate.

All subjects provided written informed consent according to the Helsinki Declaration of 1975. The local Institutional Review Board approved the protocol (Comitato Etico Regionale Liguria 278/2019 DB id 4673; 29 April 2020).

### Image acquisition and visual assessment

MRI scans were acquired on a 3-T scanner (MAGNETOM Prisma, Siemens Healthineers) equipped with a 64-channel multiarray receiver coil. Each scan included a “Magnetization Prepared two Rapid Acquisition Gradient Echo” research application sequence (“Compressed Sensing MP(2)RAGE”, Compressed sensing magnetization prepared two rapid acquisition gradient-echo, CS-MP2RAGE, Work-In-Progress #019, Siemens Healthcare) acquired with 1-mm^3^ isotropic resolution and 4.6× compressed sensing acceleration [13] (repetition time = 5,000 ms, echo time = 2.9 ms, inversion time 1 = 700 ms, inversion time 2 = 2,500 ms, flip angle 1 = 4°, flip angle 2 = 5°). In-line output, available directly on the scanner, included an intensity-homogeneous denoised three-dimensional (3D) T1-weighted image (default denoising factor = 5) [14] and a qT1 map intrinsically coregistered to it [15], as both originate from the combination of the same acquisition. A 3D turbo spin-echo T2-weighted fluid-attenuated inversion-recovery (FLAIR) sequence was acquired (1-mm^3^ isotropic resolution, repetition time = 4,500 ms, echo time = 383 ms, inversion time = 1,800 ms, flip angle = 120°). Deep white matter (WM) hyperintensities were visually assessed on T2-weighted fluid-attenuated inversion-recovery images using the Fazekas scale (0–3) [16], with higher scores indicating greater lesion burden; images were scored by a neuroradiologist with six years of experience in the field (L.G.). Group differences in Fazekas score distribution were assessed using the χ² test.

Amyloid-PET scans were acquired in a subset of patients with a PET/computed tomography–CT system (BioGraph mCT 16, Siemens Healthineers) following the European Association of Nuclear Medicine and the Society of Nuclear Medicine guidelines [17], reconstructed on a 256 × 256 matrix (1.33 × 1.33 × 2.00 mm^3^ voxel size), and employing [^18^F]-Florbetaben or [^18^F]-Flutemetamol as tracers; doses ranged between 185 MBq and 370 MBq according to the manufacturer’s recommendations. Two nuclear medicine specialists visually assessed PET positivity.

### Association between volume, qT1, and cognition in the hippocampus

CS-MP2RAGE 3D T1w denoised images were processed with FreeSurfer (Martinos Center for Biomedical Imaging, Massachusetts General Hospital) version 6.0 to obtain segmentation masks of the hippocampi [18]. Hippocampal qT1 values were analyzed at a regional level using a linear mixed-effects model to examine group differences (AD *versus* HCs), adjusted for volume, sex, age, and side, accounting for subject random effects. The same analysis was repeated, subdividing AD patients with a median split on MMSE scores.

### Voxel-wise comparison between AD and HCs

To compare the voxel-wise distribution of atrophy and qT1 values between patients and controls, CS-MP2RAGE 3D T1w denoised images were processed in Statistical Parametric Mapping 12 (SPM 12, Wellcome Centre for Human Neuroimaging, University College London) running in Matlab 2023b (The MathWorks, Inc.) with the Dartel tool [19] to obtain a common spatial template. qT1 maps were coregistered to the template with the same transformation computed for 3D T1w images and smoothed using a 10-mm Gaussian kernel. Tissue probability maps for GM (c1), WM (c2), and CSF (c3) were also computed inside the SPM 12 pipeline.

Group differences were assessed voxel-wise in SPM 12 with a linear model accounting for age, sex, and brain parenchymal fraction (the latter only for qT1). Brain parenchymal fraction was calculated as the ratio between total brain volume and estimated total intracranial volume, as derived from FreeSurfer.

Analyses were restricted to a cortico-limbic mask derived from thresholding the probabilistic segmentation of the GM at 0.5 (*i.e*., voxels with > 50% probability of being GM, therefore a higher probability than that of all other classes combined), followed by manual exclusion of infratentorial and deep structures. The voxel-level significance threshold was set at *p* < 0.05 corrected for multiple comparisons using the false discovery rate method, with a minimum cluster size of 50 voxels.

### Voxel-level correlation between qT1 and amyloid-PET

In subjects with available amyloid-PET scans, we evaluated the voxel-level relationship between qT1 and standardized uptake value ratios (SUVr) normalized to the cerebellum. Normalized PET scans were coregistered to the template and smoothed with the same 10-mm kernel used for qT1 maps. Then, we extracted the values of SUVr, qT1, and SPM-derived tissue probability values for CSF and WM for each voxel within the cortico-limbic mask defined in the previous analysis, using the Functional MRI of the Brain (FMRIB) Software Library—FSL (FMRIB Centre, University of Oxford). Partial volume effects involving residual mixed tissue components were factored out employing a linear mixed-effects model to estimate the fixed effect of SUVr on qT1, controlling for CSF and WM probability, which were included as continuous covariates in the model. Tracer type and the time interval between PET and MRI were also included as covariates to account for their potential confounding effects on SUVr and its relationship with qT1. Subjects were included as a random intercept to account for the non-independence of voxel-level observations within individuals. Given the large number of observations in voxel-level data, we reported effect sizes along with *p*-values of the model’s fixed effects. Effect sizes are reported as Cohen’s d, which represents the standardized difference between variables in units of standard deviation, conventionally considered small = 0.2, medium = 0.5, and large = 0.8 [20].

A schematic representation of the Methods workflow is provided in Supplementary Fig. S1.

## Results

### Demographic and clinical features

The AD group included 33 subjects (age 69.3 ± 8.2 years, mean ± standard deviation; 18 females), while 22 subjects were enrolled as HCs (age 70.5 ± 13.3 years; 15 females). No significant differences were found between these two groups for age (*p* = 0.684), sex (*p* = 0.312), and Fazekas score (*p* = 0.160).

Amyloid-PET was acquired for 16 patients (7 with [^18^F]-Florbetaben, 9 with [^18^F]-Flutemetamol); visual assessment showed full agreement in all cases. The PET-MRI interval was on average -7.8 ± 14.1 months (negative values indicate that PET was performed before MRI). Sample characteristics are summarized in Table 1.

**Table 1:** Sample characteristics for the SPM 12 analysis and voxel-level qT1-PET correlation analysis

	AD	HC
**A.** **Sample characteristics for the SPM 12 analysis**		
*N*	33	22
Age (years)	69.3 ± 8.2	70.5 ± 13.3
Sex (F/M)	18/15	15/7
BPF	0.664 ± 0.033	0.722 ± 0.044
Fazekas score (0/1/2/3)	3/21/4/5	5/15/2/0
**B.** **Sample characteristics for the voxel-level qT1-PET correlation analysis**		
*N*	16	–
Age (years)	68.2 ± 7.23	–
Sex (F/M)	7/9	–
Fazekas score (0/1/2/3)	2/10/2/2	–
qT1(ms)	1,562.838 ± 262.145	–
SUVr	1.373 ± 0.280	–
WMp	0.215 ± 0.142	–
CSFp	0.288 ± 0.153	–
Tracer ([^18^F]-Florbetaben/[^18^F]-Flutemetamol)	7/9	–
PET-MRI interval (months)	-7.8 ± 14.1^*^	–

Values are expressed as mean ± standard deviation, except for sex, Fazekas score, and tracer, which are reported as counts. In panel B, qT1, SUVr, WMp, and CSFp are reported as mean ± standard deviation within the cortico-limbic mask used for the analysis

*BPF* Brain parenchymal fraction, *CSFp* Cerebrospinal fluid partial volume fraction, *MRI* Magnetic resonance imaging, *PET* Positron emission tomography, *qT1* Quantitative T1 relaxation time, *SUVr* Standardized uptake value ratio, *WMp* White matter partial volume fraction

^*^ If the value is negative, PET was performed before MRI

**Fig. 1:**
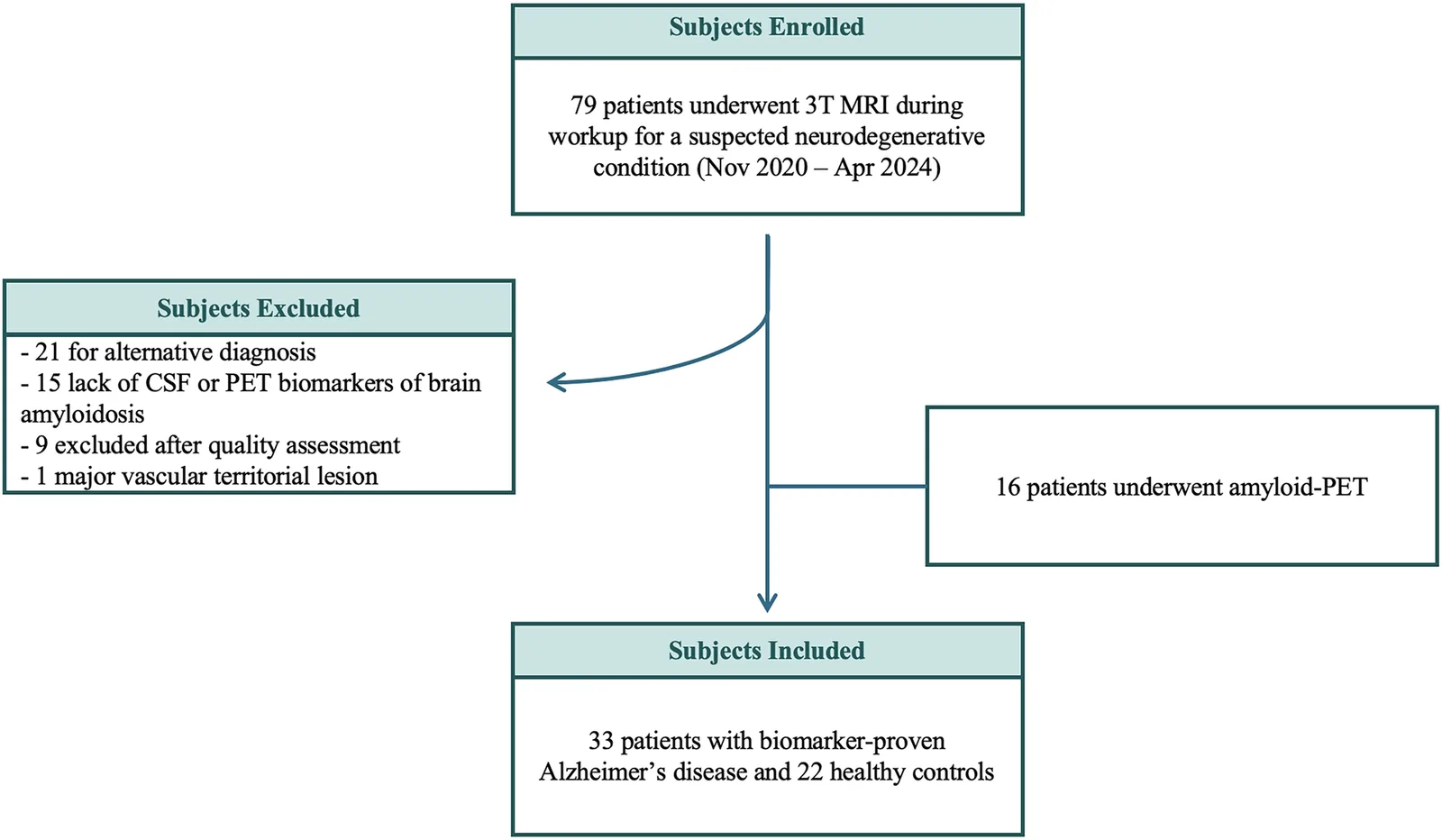
Study flow diagram illustrating participant selection. Alternative diagnoses included Lewy body dementia, frontotemporal lobar degeneration, and vascular cognitive impairment. Among the subjects excluded after quality assessment, four were excluded due to motion artifacts, three for incomplete protocol, and two were excluded because of magnetic susceptibility artifacts generated by non-removable prosthetic dental materials

Figure 1 shows the flow diagram of participant selection, exclusion, and final inclusion in the study.

### Regional analysis of the hippocampus stratified for MMSE

**Fig. 2:**
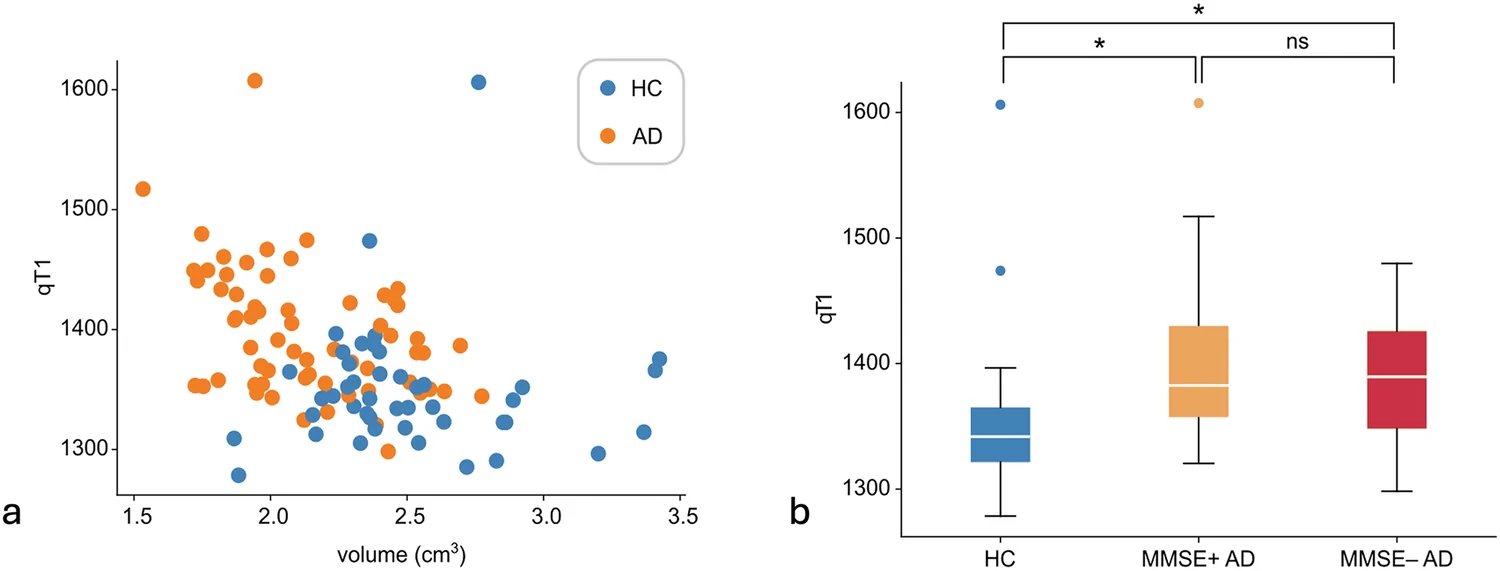
Regional analysis in the hippocampus. **a** Scatter plot of volume against regional qT1 in the hippocampi in the HCs and AD groups. **b** Box plot reporting the distribution of regional qT1 values in the hippocampi within the HCs, MMSE + AD, and MMSE- AD groups; * Significant differences. AD, Alzheimer disease; HCs, Healthy controls; MMSE, Mini-mental state examination; qT1, Quantitative T1 relaxation time

Regional analyses of qT1 values in the hippocampi revealed a significant group difference, with longer times in AD as compared to HCs (1,398 ± 53.0 ms *versus* 1,349 ± 53.4 ms, *p* = 0.002; Fig. 2a).

In the AD group, the MMSE score was 23.8 ± 3.42, with a median of 25, which was used as the cutoff to stratify patients into MMSE-low (< 25; 1,391 ± 48.2 ms) and MMSE-high (≥ 25; 1,400 ± 56.6 ms) subgroups. No significant differences in hippocampal qT1 were found between AD patients stratified for MMSE (*p* = 0.305; Fig. 2b).

### Voxel-based analyses

**Fig. 3:**
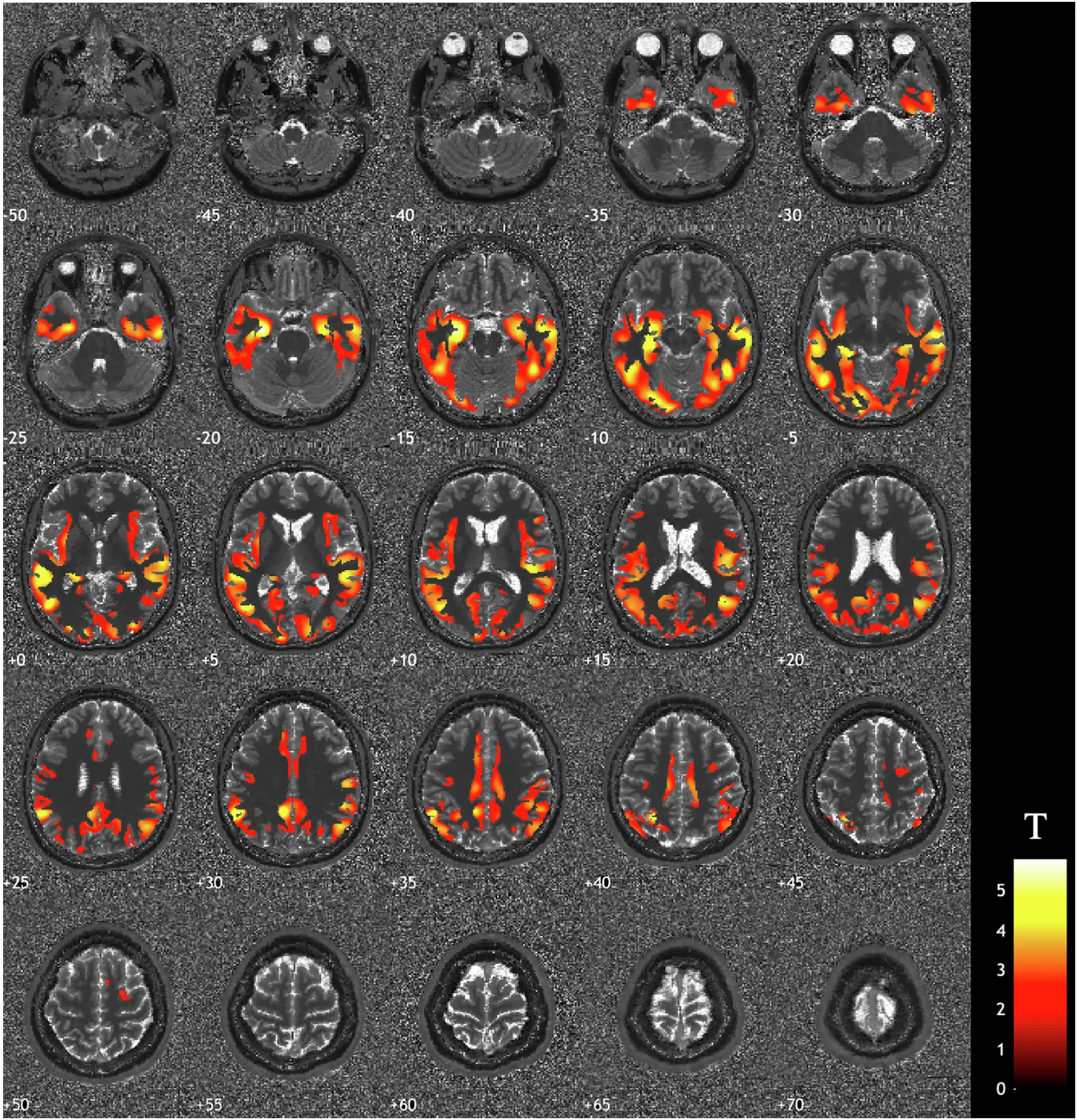
Cortical voxels with increased qT1 in the voxel-wise SPM 12 group comparison. Cortical areas showing increased qT1 value in AD, as identified through the SPM 12 analysis, overlaid on the T1 map of a healthy subject; colors indicate T-statistic values. The threshold was set at *p* < 0.05 FDR-corrected for multiple comparisons, *k* = 50. AD, Alzheimer's disease; FDR, False discovery rate; qT1, Quantitative T1 relaxation time

In voxel-wise SPM 12 analyses, AD subjects demonstrated widespread significant atrophy across the brain compared to HCs in the whole cortical mantle. Conversely, they showed a more focal distribution of increased qT1 values compared to HCs, mainly in the mesial temporal lobe structures and posterior temporal-parietal-occipital cortices (1,448 ± 55.7 ms *versus* 1,344 ± 51.6 ms; Fig. 3 and Table 2).

**Table 2:** SPM 12 voxel-wise group differences between HCs and AD

Peak *p* (FDRc)	Peak *T*	Peak equivZ	Peak *p* (unc)	*x*, *y*, *z* {mm}	Peak location (neuromorphometrics)
0.002	5.79	5.04	< 0.001	-27, -12, -18	Left hippocampus
0.003	5.20	4.63	< 0.001	-51, -50, 32	Left supramarginal gyrus
0.003	5.09	4.55	< 0.001	44, -51, -12	Right inferior temporal gyrus
0.007	3.57	3.35	< 0.001	-10, -24, 36	Left middle cingulate gyrus
0.008	3.52	3.31	< 0.001	-9, 3, 38	Left middle cingulate gyrus
0.011	3.25	3.08	0.001	-9, 27, 30	Left superior frontal gyrus
0.017	2.93	2.80	0.003	52, -8, 18	Right central operculum
0.035	2.40	2.32	0.010	51, -12, 27	Right postcentral gyrus
0.018	2.90	2.77	0.003	46, 4, 14	Right central operculum
0.020	2.79	2.68	0.004	21, -9, 48	Right precentral gyrus
0.031	2.49	2.41	0.008	32, -6, 40	Right precentral gyrus
0.025	2.65	2.55	0.005	-42, 22, 15	Left middle frontal gyrus

Cortical voxels showing increased qT1 values as identified with the SPM 12 analysis, labeled according to Neuromorphometrics (*p* < 0.05 FDRc, *k* = 50)

*FDRc* False discovery rate corrected

**Fig. 4:**
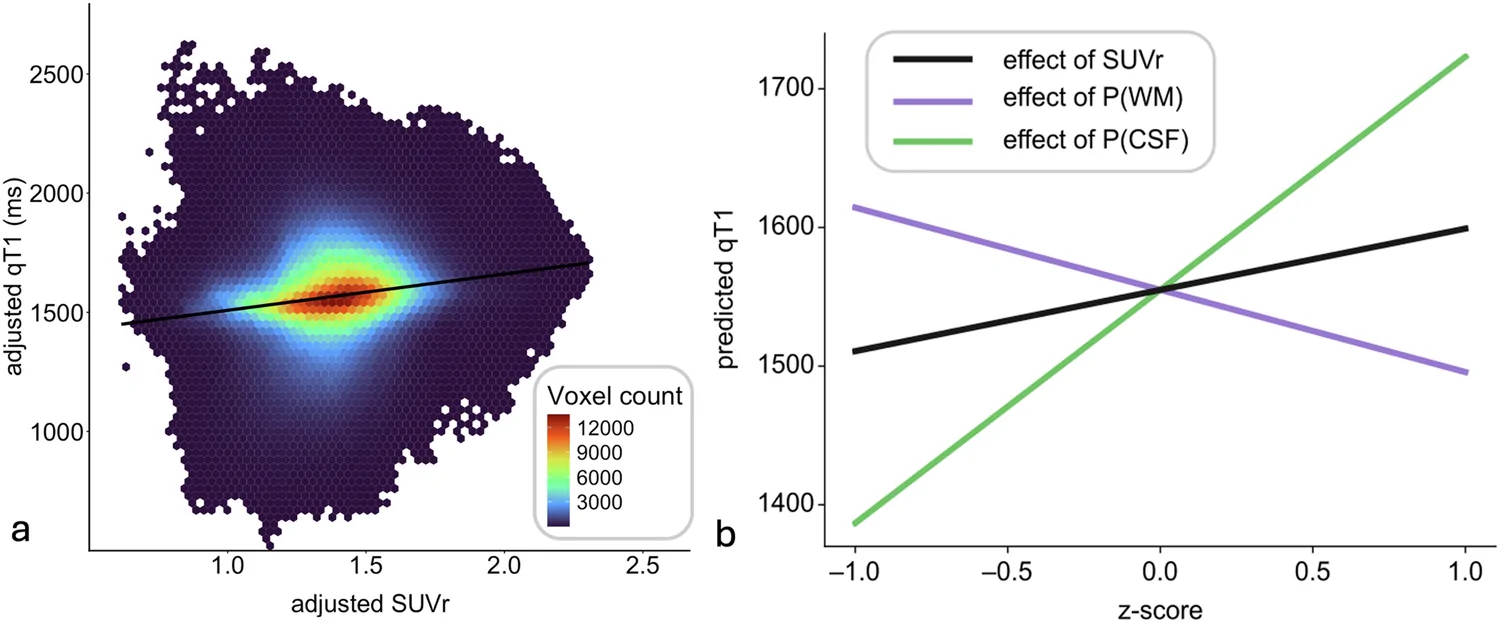
Voxel-level qT1-SUVr association. **a** The association between qT1 and amyloid-PET SUVr is visualized using a hexbin plot: the qT1-SUVr plane is divided into hexagonal bins, each colored according to the number of observations within the bin. Low-density peripheral regions exhibit increased noise and dispersion, while the high-density central cluster demonstrates a positive trend. qT1 and SUVr values are adjusted for subject-level variability, WM partial volume, and CSF partial volume. **b** Mixed-effect model estimates of qT1 as a function of WM probability, CSF probability, and SUVr. Predicted qT1 values are plotted as a function of *z*-scored values for each variable. CSF, Cerebrospinal fluid; PET, Positron emission tomography; qT1, Quantitative T1 relaxation time; SUVr, Standard uptake values ratio; WM, White matter

In the voxel-level linear mixed effect model, correcting for the partial volume effects of WM and CSF, a subtle yet consistent positive association emerged between qT1 and SUVr (SUVr *p* < 0.001, *d* = 0.223; WM *p* < 0.001, *d* = -0.313; CSF *p* < 0.001, *d* = 0.863; Fig. 4a, b). The PET-MRI time interval and the tracer used had a significant impact (*p* < 0.001 for both), but with a negligible effect size (*d* = 0.002 per month and *d* = 0.025, respectively). Supplementary Materials provide additional details on the voxel-based models’ variables and their interdependencies.

## Discussion

In this work, we demonstrate a significant qT1 increase in the cortico-limbic GM of subjects affected by AD compared to HCs. We also provide proof of an existing relationship between the qT1 increase and amyloid-PET SUVr at a voxel level. These results cannot fully elucidate the physical mechanisms underlying qT1 modifications, which can represent the result of multiple pathological pathways and tissue changes associated with amyloid presence in AD.

Firstly, tissue water content is a major driver of qT1 values. Therefore, the observed qT1 elongation may be the expression of active neuroinflammation, along with hydration water of soluble Aβ species, and more complex hydration processes that result in the retention of water molecules within amyloid plaques [21]. Indeed, an increase in cortical thickness, with associated higher mean diffusivity values, has been previously described in the preclinical stages of AD, which suggests expansion of the extracellular space and aligns with this hypothesis [22].

Conversely, the pattern of cortical qT1 alteration does not precisely align with the expected distribution of amyloid deposition [23]. However, in posterior cortical areas, amyloid and regional neurodegeneration can significantly overlap, possibly increasing the consistency of qT1 group differences.

An increase in qT1 may also result from a decrease in myelin content. A study by Dean et al showed that core CSF AD biomarkers are associated with lower longitudinal relaxation rates in the WM in AD, likely reflecting myelin loss [24], and similar changes could involve intracortical myelinated fibers.

The lack of an effect from the subdivision based on the MMSE is consistent with evidence that clinical severity is more closely associated with metrics of neurodegeneration [25] while other quantitative parameters, including microstructural properties and amyloid burden, often fail to show a straightforward association with cognition [26–28].

A key missing element for the interpretation of qT1 changes in AD is the availability of longitudinal data. Within-subject longitudinal variations may offer greater sensitivity to amyloid-related effects than between-subject differences, as in this framework, the influence of inter-individual variability and subject-specific confounding microstructural factors could be reduced, enhancing the ability of qT1 to capture changes associated with amyloid dynamics. If supported by longitudinal evidence, qT1 might play a role in monitoring disease progression or treatment-related changes over time. MRI is already incorporated into the longitudinal monitoring of patients undergoing amyloid-targeting therapies, primarily for safety assessment (*e.g*., amyloid-related imaging abnormalities, ARIA) [29, 30]. At the same time, current evidence suggests a treatment-to-clean approach where drug discontinuation should be considered based on reductions in amyloid burden, which is assessed with longitudinal amyloid-PET monitoring [31–33]. In this context, the development of MRI-derived surrogate markers reflecting amyloid burden is of particular interest, as they could provide complementary information on amyloid-targeting therapies’ effects and potentially contribute to optimizing the timing and frequency of amyloid-PET evaluations, with advantages in terms of reduced invasiveness and cost.

The CS-MP2RAGE implementation used in this work enables substantial reductions in acquisition time while preserving quantitative reliability. Previous studies have demonstrated that CS-MP2RAGE provides qT1 estimates comparable to conventional acquisitions, with good agreement in morphometric measures and high scan-rescan reproducibility [13, 34–36]. The use of CS allowed whole-brain coverage at 1-mm^3^ isotropic resolution in approximately 3 min and 40 s, compared to over 8 min for standard MP2RAGE protocols. This represents a relevant advantage in clinical settings, particularly in neurodegenerative populations, where shorter acquisition times may improve patient compliance.

This study has some limitations due to its exploratory nature. A potential bias derives from the difference in spatial resolution between MRI and PET images. To address this, we applied a statistical model accounting for the probability of WM and CSF inclusion within each voxel. It should be noted that partial volume effects with surrounding tissues would be expected to produce the opposite trend, as CSF shows negligible PET uptake and markedly higher qT1 values compared to cortical GM. In contrast, WM exhibits substantially lower qT1 values than GM, and the region showing the highest tracer uptake at amyloid-PET is typically considered to be embedded between the cortex and the immediately subjacent WM layer, a pattern likely reflecting a combination of factors, including mainly the limited spatial resolution of PET imaging resulting in blurring of the WM-GM border, and the physiologic intrinsic tracer uptake of the WM [37].

The retrospective nature and the small sample size limit the generalizability of our findings. To increase the reliability of the results, we focused on subjects with a solid diagnostic profile, and we applied a robust statistical analysis to control for the impact of atrophy at the individual level.

In conclusion, this study demonstrates cortico-limbic qT1 alterations in AD and their association with amyloid-PET SUVr distribution, supporting qT1 mapping as a promising and noninvasive imaging biomarker of AD pathology. Further research is warranted to better characterize the pathological substrate of the observed association between qT1 and amyloid-PET SUVr and to validate this metric as a clinically feasible tool for detecting and monitoring microstructural changes in neurodegeneration.

## Supplementary information

**Additional file 1: Supplementary Fig. S1** Methods workflow. Schematic overview of imaging acquisition and processing. All subjects underwent 3-T MRI with CS-MP2RAGE, generating intrinsically coregistered T1-w images and quantitative T1 maps (qT1); a subgroup of AD patients also underwent amyloid-PET. c1, c2, and c3 represent probabilistic maps of GM, WM, and CSF, respectively, obtained from SPM 12. Red boxes indicate the endpoint statistical analyses for each pipeline. **Supplementary Fig. S2** Voxel-level interdependencies among qT1, SUVr, WMp, and CSFp. On the left, a scatterplot matrix showing pairwise relationships among the variables in the voxel-level qT1-PET linear mixed-effects model. Scatter plots display the relationships between qT1, SUVr, WMp, and CSFp across all sampled voxels in an example AD subject. The associations depicted are uncorrected and reflect raw relationships, emphasizing the importance of accounting for different tissue composition to analyze quantitative trends properly. On the right, an illustration exemplifying the opposite signal gradient in qT1 (upper image) and SUVr maps (lower image), which is expected on an anatomical basis. Notably, the SUVr gradient is driven by the lipophilic nature of amyloid tracers and their affinity for myelin even in the absence of amyloid deposits. As expected, CSF partial volume has a positive effect on qT1 values and a negative effect on SUVr, while WM partial volume shows an opposite pattern. The raw relationship between qT1 and SUVr mainly reflects these trends.

## Data Availability

The data that support the findings of this study are available on reasonable request from the corresponding author. Imaging data are not publicly available due to privacy or ethical restrictions.

## Abbreviations

3D: Three-dimensional
AD: Alzheimer disease
CSF: Cerebrospinal fluid
CS-MP2RAGE: Compressed sensing magnetization prepared two rapid acquisition gradient-echo
GM: Gray matter
HCs: Healthy controls
MMSE: Mini-mental state examination
MRI: Magnetic resonance imaging
PET: Positron emission tomography
qT1: Quantitative T1 relaxation time
SPM: Statistical parametric mapping
SUVr: Standard uptake values ratio
WM: White matter
