# Supplementary material for: T1 mapping in Alzheimer’s disease: a quantitative MRI study with amyloid-PET correlation
Source: Eur Radiol Exp. 2026 Sep 21;10:137. doi: 10.1186/s41747-026-00792-1 (PMC13593977; doi:10.1186/s41747-026-00792-1)
Supplement: Supplementary file 1 [file 41747_2026_792_MOESM1_ESM.pdf]

T1 mapping in Alzheimer disease: a quantitative MRI study with amyloid-PET correlation

ELECTRONIC SUPPLEMENTARY MATERIAL

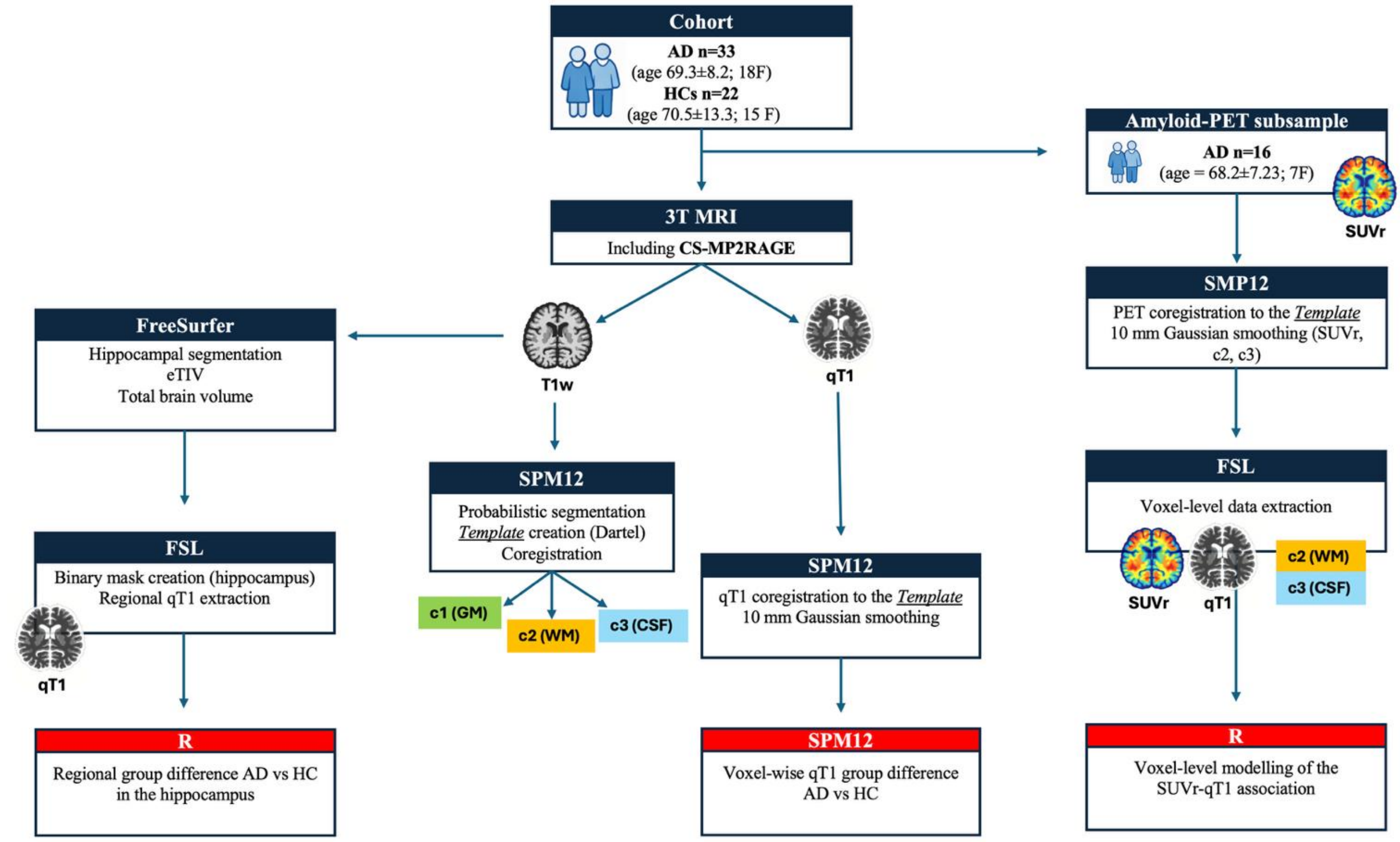

Supplementary Fig. S1 Methods workflow. Schematic overview of imaging acquisition and processing. All subjects underwent 3T MRI with CS-MP2RAGE, generating intrinsically coregistered T1-w images and quantitative T1 maps (qT1); a subgroup of AD patients also underwent amyloid-PET. c1, c2, and c3 represent probabilistic maps of GM, WM, and CSF, respectively, obtained from SPM 12. Red boxes indicate the endpoint statistical analyses for each pipeline. *AD* Alzheimer disease, *CSF* Cerebrospinal fluid, *CS-MP2RAGE* Compressed sensing magnetization-prepared two rapid acquisition gradient echo, *eTIV* Estimated total intracranial volume, *FSL* FMRIB Software Library, *GM* Gray matter, *HCs* Healthy controls, *MRI* Magnetic resonance imaging, *PET* Positron emission tomography, *qT1* Quantitative T1, *SPM* Statistical Parametric Mapping, *SUVr* Standardized uptake value ratio, *T1w* T1-weighted, *WM* White matter.

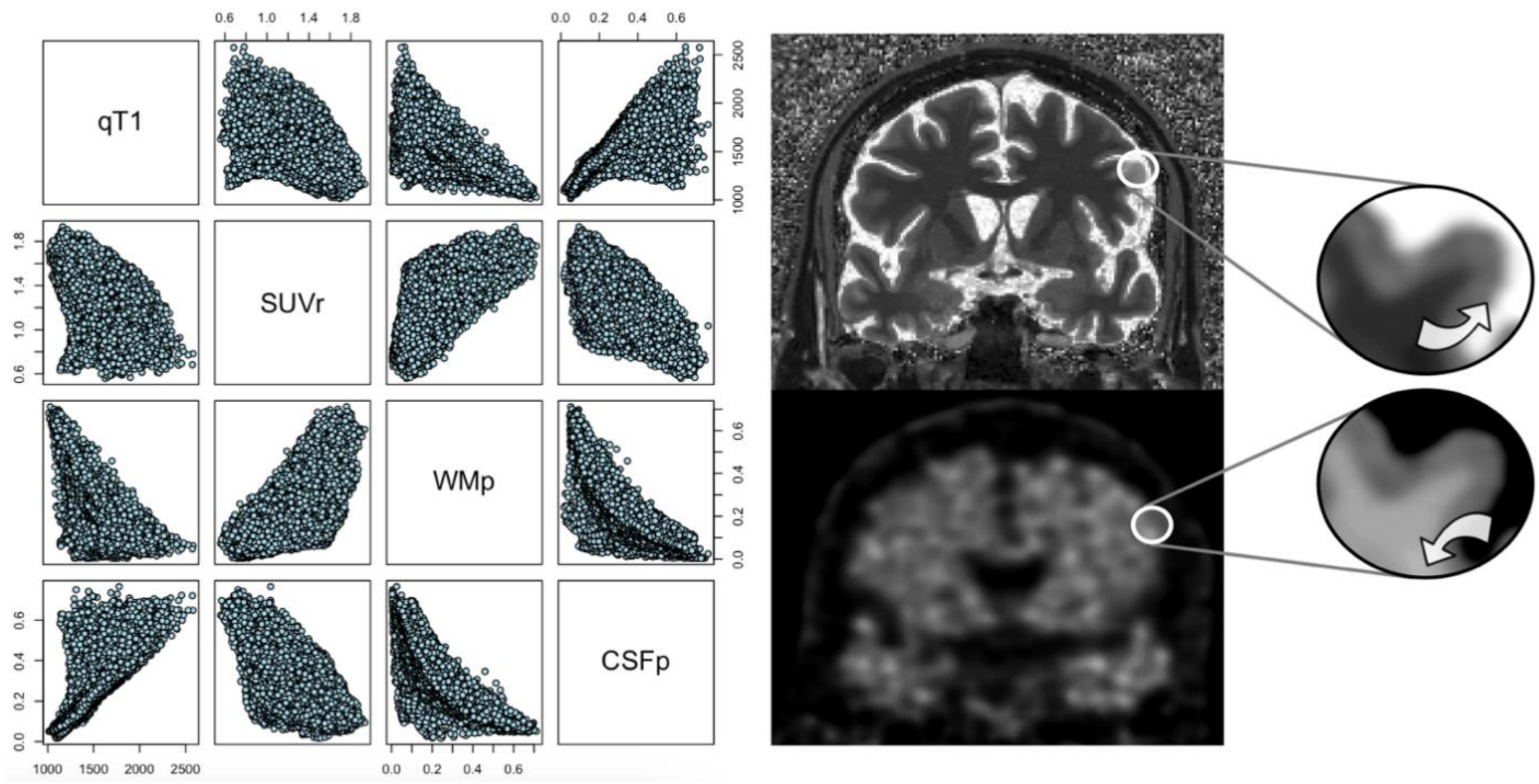

Supplementary Fig. S2 Voxel-level interdependencies among qT1, SUVr, WMp, and CSFp. On the left, a scatterplot matrix showing pairwise relationships among the variables in the voxel-level qT1-PET linear mixed-effects model. Scatter plots display the relationships between qT1, SUVr, WMp, and CSFp across all sampled voxels in an example AD subject. The associations depicted are uncorrected and reflect raw relationships, emphasizing the importance of accounting for different tissue composition to analyse quantitative trends properly. On the right, an illustration exemplifying the opposite signal gradient in qT1 (upper image) and SUVr maps (lower image), which is expected on an anatomical basis. Notably, the SUVr gradient is driven by the lipophilic nature of amyloid tracers and their affinity for myelin even in the absence of amyloid deposits.
As expected, CSF partial volume has a positive effect on qT1 values and a negative effect on SUVr, while WM partial volume shows an opposite pattern. The raw relationship between qT1 and SUVr mainly reflects these trends. *CSFp* Cerebrospinal fluid probability, *qT1* Quantitative T1, *SUVr* Standardized uptake value ratio, *WMp* White matter probability.
